# An Automated and Miniaturized Rotating-Disk Device for Rapid Nucleic Acid Extraction

**DOI:** 10.3390/mi10030204

**Published:** 2019-03-22

**Authors:** Rui Tong, Lijuan Zhang, Chuandeng Hu, Xuee Chen, Qi Song, Kai Lou, Xin Tang, Yongsheng Chen, Xiuqing Gong, Yibo Gao, Weijia Wen

**Affiliations:** 1 The Nano Science and Technology (NSNT) Program, The Hong Kong University of Science and Technology, Clear Water Bay, Kowloon, Hong Kong; rtongaa@connect.ust.hk (R.T.); xchendi@connect.ust.hk (X.C.); 2 Shenzhen Shineway Hi-Tech Co., Ltd., Shenzhen 518112, China; zlj@swtech.me (L.Z.); lk@swtech.me (K.L.); 3 Department of Physics, The Hong Kong University of Science and Technology, Clear Water Bay, Kowloon, Hong Kong; chuae@connect.ust.hk; 4 Guangzhou HKUST Fok Ying Tung Research Institute, Nansha, Guangzhou 511458, China; songqi2712@gmail.com (Q.S.); xintang@ust.hk (X.T.); 5 Department of Ocean Science, The Hong Kong University of Science and Technology, Clear Water Bay, Kowloon, Hong Kong; yschen@connect.ust.hk; 6 Materials Genome Institute, University of Shanghai, Shanghai 200444, China; gongxiuqing@shu.edu.cn

**Keywords:** sample preparation, nucleic acid, DNA, RNA

## Abstract

The result of molecular diagnostic and detection greatly dependent on the quality and integrity of the isolated nucleic acid. In this work, we developed an automated miniaturized nucleic acid extraction device based on magnetic beads method, consisting of four components including a sample processing disc and its associated rotary power output mechanism, a pipetting module, a magnet module and an external central controller to enable a customizable and automated robust nucleic acid sample preparation. The extracted nucleic acid using 293T cells were verified using real-time polymerase chain reaction (PCR) and the data implies a comparable efficiency to a manual process, with the advantages of performing a flexible, time-saving (~10 min), and simple nucleic acid sample preparation.

## 1. Introduction

With the rapid development of molecular biology technology in recent years, molecular diagnostic and detection technologies represented by nucleic acid hybridization, nucleic acid amplification and nucleic acid sequence analysis have become increasingly significant in many fields. However, the fundamental challenge facing all modern molecular biology detection techniques, such as polymerase chain reaction (PCR), high-throughput sequencing, etc. is how to promptly and efficiently separate and extract the required genomic nucleic acid from complex and diverse biological samples, since the quality and integrity (state of degradation) of the isolated nucleic acid directly affects the subsequent experimental results [1]. At present, researchers all over the world have made many breakthroughs in the technology of nucleic acid separation and extraction. 

Nucleic acids are broadly classified into deoxyribonucleic acid (DNA) and ribonucleic acid (RNA). Ever since it was first discovered in 1869, many researchers have made unremitting efforts in the extraction of nucleic acids, and have improved various materials and reagents for nucleic acid extraction. Milestone research findings includes: phenol extraction technique [2], sodium dodecyl sulfate (SDS) method [3], and acid guanidinium phenolchoroform (AGPC) extraction technique [4,5]. Numerous well-known biological reagent companies have developed various nucleic acid extraction kits based on these conventional nucleic acid extraction methods for the separation and extraction of DNA and RNA from a wide variety of tissue samples. The conventional methods of nucleic acid extraction often include precipitation and centrifugation, which require an extensive number of steps, and thus are complicated, time-consuming (requiring up to 3 h, and much longer if incubated overnight) [6], and difficult to achieve miniaturized automation. Most of the methods require operators to be in direct contact with toxic chemical reagents. Therefore, with the rapid development of molecular biology and polymer materials science, the conventional method of separating and extracting nucleic acids from liquid phase systems has been gradually replaced by new methods based on solid phase adsorbate carriers [7,8]. Such emerging nucleic acid separation and extraction methods mainly include: Glass particles method [9], silica matrices method [10,11], anion exchange method [12], and magnetic beads-based extraction method [13]. Regardless of which method is used to separate and extract nucleic acids, in general, the operation steps of such methods can be mainly divided into four parts [7,8,14]. The first part is to use the lysis to promote cell disruption and release the nucleic acids. The second part is to specifically adsorb the released nucleic acids on a specific carrier, with this specific carrier has strong affinity and adsorption only for nucleic acids, but has no affinity for other biochemical components such as proteins, polysaccharides, and lipids. The third part is to wash with a specific washing buffer to remove non-nucleic acid impurities, and the last part is to elute the nucleic acid adsorbed on the specified carrier to obtain purified nucleic acid [15]. 

The extraction of nucleic acids by the spin column-based method has been widely used, and most of the plasmid DNA extraction kits on the market have been developed based on the spin column-based method. The method adopts a special silicon matrix adsorption material, which is characterized as follows: In the presence of a high hydrochloric acid buffer, the DNA can be specifically adsorbed, the impurities can be removed with a series of washing steps, and the low-salt alkaline buffer can elute the DNA bound to the adsorption column [14]. However, the disadvantage of this method is that the sample required is large, thus consuming a lot of samples. Furthermore, the application of this method on some rare samples is greatly limited. At the same time, the spin column method requires repeated centrifugation during the process, which is not suitable for a high-throughput, automated operation. Especially in the field of genetic diagnosis, monitoring and control of sudden outbreaks, the use of the spin column-based method to extract nucleic acids requires a large number of operators and equipment to meet the demand. Since the 1990s, due to various deficiencies in the spin column method, in order to adapt to the high-throughput, high-sensitivity, and automated operation requirements of modern molecular biology testing experiments, the method of extracting nucleic acids using magnetic beads emerged [16]. This method is the perfect combination of nanotechnology and biotechnology since magnetic beads are high-affinity composite magnetic microspheres (typically 1 to 100 nm) formed by combining an inorganic magnetic particle with a polymeric material. This method advantages that other nucleic acid extraction methods cannot match, which are mainly reflected in: (1) It can realize high-throughput operation and automation; (2) The operation is simple and time-saving, the entire extraction process consists only four steps, and the whole process can be completed within 40 min; (3) It is safe and non-toxic; (4) The specific binding of magnetic beads and nucleic acid makes the extracted nucleic acid high in purity and concentration; (5) Low in cost and can be applied in a wide number of applications. Since magnetic bead synthesis uses low-cost inorganic and organic raw materials, no special equipment is required, which makes the final synthesis, research, and development costs very low [17,18]. A major improvement in nucleic acid extraction by magnetic beads is the use of a high-affinity composite magnetic microsphere, formed by the combination of inorganic magnetic particles and polymer materials. Because of its many properties of polymer microspheres and magnetic particles, it is uniformly and stably dispersed in the solution without an external magnetic field, and can be easily and quickly separated once the external magnetic field is added. 

Microfluidic technology originated from the concept of a micro-electro-mechanical system based on MEMS technology, as proposed by Manz et al. in the early 1990s [19]. The purpose of this system is to transfer functions of the lab onto portable devices, and even chips, through miniaturization and integration of chemical analysis systems. The core of this technology is microfluidic chips, in which a series of microchannels are fabricated on a small chip. Through the manipulation and control of the microfluids in the microchannel, the entire chemical and biological laboratory functions are realized. Several microfluidic chip nucleic acid extraction techniques have been reported to isolate target cells from blood samples by using micromachined ‘weir-type’ filters [20], pore filters [21], or pathogen-specific immunomagnetic beads [22]. The captured target cells are introduced into a PCR reaction chamber on a chip, and the DNA is released by cell thermal lysis. However, since the mixture present in the cell debris may inhibit the PCR process, most of the microfluidic chips reported for DNA extraction require preliminary off-chip sample processing steps. For DNA extraction on the chip, the most common method currently used is to extract DNA from cell lysates using magnetic beads coated with silica or functional groups (carboxy [22], amine [23], biotin [24], nucleotide probes [25]). In addition, there have been successful studies on dielectrophoretic trapping [26] and isotachophoresis [27] on DNA purification microfluidic chips.

Although most developments in the field of molecular diagnostics have focused on improving methods for detecting and identifying disease-related target analytes, less attention has been devoted to developing systems for purifying samples. Our goal is to develop a customizable, automated, and miniaturized system for nucleic acid sample preparation based on the magnetic beads method. In this work, we developed an automated miniaturized nucleic acid extraction device consisting of four components including a sample processing disc and its associated rotary power output mechanism, a pipetting module, a magnet module, and an external central controller. The device was designed discreetly to ensure its portability (220 × 165 × 210 mm dimension and 3 kg weight), efficiency, and easiness to operate. Our platform integrates the functions of lysis, binding, washing and elution, and can be customized according to different extraction protocols to meet various user demands. We analyzed the performance of the device using 293T cells, the extracted nucleic acids were detected using real-time PCR and verified by electrophoresis. Manual process of nucleic acid extraction usually requires 40 to 50 min, and the previously reported microchip-based DNA extraction costs 12 min [28] to 15 min [22], our device further curtailed the process duration within 10 min. The commercialized automated nucleic acid extraction instruments (e.g., QIAcube from QIAGEN, Hilden, Germany) and liquid handling robots or platforms (e.g., QIAgility from QIAGEN) have similar functions as our device but with a much bulkier size and heavier weight (QIAcube is 71.5 kg and QIAgility is 41 kg). The small dimension, light weight, and rapid processing time makes our device suitable for future use in point-of-care testing.

## 2. Materials and Methods 

### 2.1. Design Concept

**Figure 1:**
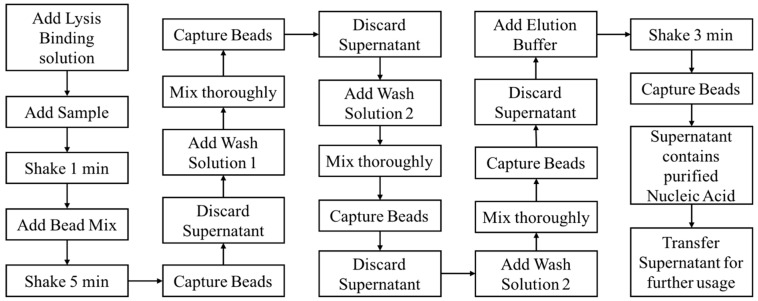
Protocol for nucleic acid extraction using the magnetic bead method.

Compared with the conventional nucleic acid extraction method, magnetic beads extraction and purification of nucleic acid has incomparable advantages. The experiment process is simple, easy to operate, and can save time since the time-consuming centrifugation and precipitation steps required in the conventional methods are not required when using magnetic particles. Moreover, this system can be used on both manual and automated processes. The process is also safe and nontoxic, since it does not use the toxic reagents such as benzene and chloroform required in the conventional methods. The specific binding of magnetic particles and nucleic acid makes the extracted nucleic acid high in purity and concentration. Magnetic bead nucleic acid extraction can generally be divided into four steps: Lysis, binding, washing, and elution. Based on the process flow of the magnetic bead nucleic acid extraction as shown in Figure 1, we designed and fabricated an automated miniaturized device in realizing of the automation of all the steps and functions, thereby reducing manpower and improving purification efficiency. 

### 2.2. Configuration and Mechanism of the Device

**Figure 2:**
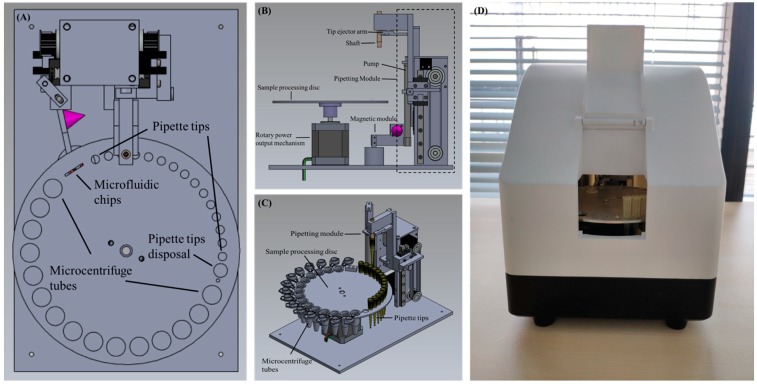
Schematic diagram of the automated miniaturized rotary sample preparation device. (**A**–**C**) Top, front, side and isometric view of the device. (**D**) Actual image of the device with all modules integrated in a portable box.

The device consists of a sample processing disc and its associated rotary power output mechanism, a pipetting module, a magnet module, and an external central controller to control the execute commands of all modules in tandem or in parallel. The schematic diagram of the device is shown in Figure 1. The sample processing disc, as shown is Figure 2A, has numerous round holes of different sizes on the outermost circumference. In particular, holes with the smallest size are for pipette tip placement, holes with the largest size are for the placement of 1.5 mL microcentrifuge tubes, and there is a medium sized hole with a circumference slightly greater than the maximum circumference of the pipette tip, which is employed for the disposal of used pipette tips and reagent waste. The rectangular hole is for placing the microfluidic chip, which is used for further PCR. The rotary power output mechanism is located underneath the sample processing disc and is connected to its center to regulate the rotating angle of the disc, as shown in Figure 2B. The pipetting module comprises of a shaft for holding pipette tips, a tip ejector arm for removal of tips, and a pump used to draw up or dispense the liquid from the disposable pipette tip. In addition, two stepping motors each connect to a slip belt (Figure 2C) are coupled to the external central controller separately to drive the shaft and the pump to move vertically relative to the horizontal plane. The magnet module includes a cone magnet (Figure 2A,B) colored in purple) to supply an external magnetic field, and a servo motor connects to the external central controller to dominate the presence and absence of the magnetic field. Furthermore, the sample processing disc, the rotary power output mechanism, the pipetting module, and the magnet module are integrated into a portable box with a dimension of 220 × 165 × 210 mm, as shown in Figure 2D, and the whole device has a weight of approximately 3 kg. Inside the portable box, a UV light controlled by the external controller is located on top to expose and decontaminate the device, therefore eliminating contamination between runs.

### 2.3. Experiment Process

**Figure 3:**
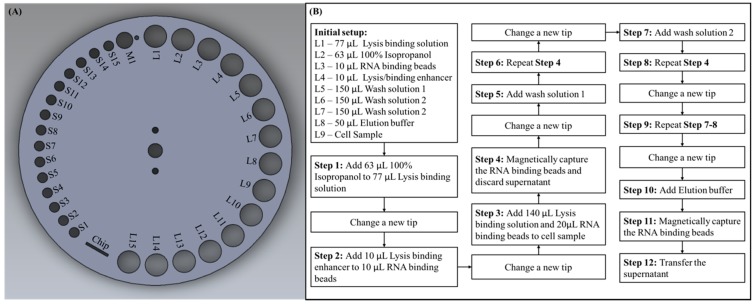
Experiment setup and procedure. (**A**) Detailed labels of each placement holes on the sample processing disc. (**B**) Process flow for automated sample preparation.

As described in the previous section, the sample processing disc has various sizes of round holes located on the outermost circumference. For experimental purposes, we assigned labels for each hole according to their sizes, as shown in Figure 3A. The smallest holes for placing pipette tips were labeled S, the medium sized hole for waste disposal was labeled M, and the largest holes for placing microcentrifuge tubes were labeled L. Prior to starting the experiment, we were required to add each reagent with a specified volume to the microcentrifuge tubes and then place them on the predetermined labeled holes, as shown in the initial setup of Figure 3B. The cell sample we used was 293T cells cultured in Dulbecco’s modified Eagle’s medium (DMEM; Gibco, Carlsbad, CA, USA) with 10% fetal bovine serum (FBS; Gibco, USA) and 1% Penicillin/Streptomycin at 37 °C in 5% CO_2_ with an initial amount of 2 × 10^6^ cells. The software program in the external central controller, which controls each module independently had been pre-set in accordance with the protocol of this experiment, and the program could be conveniently customized if using different protocols or kit. After placing all the required pipette tips and microcentrifuge tubes on the corresponding holes, the sample preparation process could be started by simply pressing the start button on the external central controller. The entire process could then be automatically conducted by the pre-set program. The detail processes ran by the device are described below, and the process flow is shown in Figure 3B. First, the sample processing disc rotated so that S1 was right underneath the shaft, the shaft moved vertically down to take in a pipette tip and moved upward after. To transfer reagent from L2 to L1, the sample processing disc rotated so that L2 was underneath the shaft, the shaft moved downward to a position where the pipette tip was merged in the reagent and the pump moved upward to aspirate the reagent into the pipette tip. The sample processing disc then rotated to L1, the shaft and pump moved downward to dispense the reagent, the pump then moved up and down repeatedly to aspirate and dispense the liquid to mix the reagents thoroughly. The used pipette tip must be changed before next step, thus the sample processing disc rotated so that M1 was underneath the shaft, and the shaft moved downward to trigger the tip ejector arm to dispose the used pipette tip and a waste container was located beneath to store used tips and reagent waste. After a new tip was introduced from S2, liquid in L4 was transferred to L3 and mixed thoroughly. Following this, the used tip was ejected through M1, a new was taken tip from S3 and liquid was transferred from L1 to L9, and then from L3 to L9 and mixed thoroughly. The magnet was then shifted to L9 to apply the magnetic field in order to magnetically capture the binding beads, with the magnetic field on, the shaft moved downward to aspirate the supernatant then discarded the used tip along with the supernatant into the waste container through M1. A new tip was taken from S4, and liquid was transferred from L5 to L9 and mixed thoroughly. Following this, the magnetic field was applied to L9 and the supernatant was aspirated and disposed. A new tip was taken from S5, and liquid was transferred from L6 to L9 and the previous steps were repeated. After this, a new tip was taken from S6 and liquid was transferred from L7 to L9 with the process described above repeated again. Until this point in the process, all the reagent additions, mixing, and washing steps required in this protocol had been completed by the system. Finally, a new tip was taken from S7 and the elution buffer located in L8 to L9 was transferred and mixed thoroughly. The used tip was then ejected through M1 and a new tip was taken from S8, a magnetic field was applied to L9, and the supernatant was aspirated, the purified RNA was also in the supernatant for further downstream applications. During this experiment, we dispensed the supernatant, which contained purified RNA in an empty microcentrifuge tube placed on L10 for additional RNA reverse transcription and PCR verification experiments. Furthermore, the sample acquired could be directly dispensed into the microfluidic chip placed on the sample processing disc for downstream experiments in the future. 

However, the process flow shown in Figure 3B is designed in accordance with the reagents and protocol we chose in our experiment. It can be easily seen that we had only occupied L1 to L9 and S1 to S8, there were still many placement holes left for use if the protocol required more reagents. Since each step of the experiment consisted of many micro steps preprogrammed in the external controller, the whole experiment process could be easily customized to meet different demands. For example, in step 3, in order to add 140 μL lysis binding solution in L1 to cell sample L9, the sample processing disc was programmed to rotate so that L1 was under the shaft, the shaft then moved vertically down until the pipette tip was merged in the reagent. Following this, the pump moved upward to aspirate the liquid into the pipette, and the shaft moved back up so that the sample processing disc could be rotated until L9 was under the shaft. Finally, the shaft moved downward and so did the pump to dispense the reagent in L9. The rotation degree of the sample processing disc, moving distance of the shaft, and the pump were all controlled by the external central controller by entering a preset value. Therefore, if the user would like to transfer reagent from L10 to L11, the user could simply program it in the external central controller. If the user only needed two washing steps, he or she can easily delete the program relating to step 9 (in Figure 3B) in the programmable controller.

### 2.4. Microchip Fabrication

**Figure 4:**
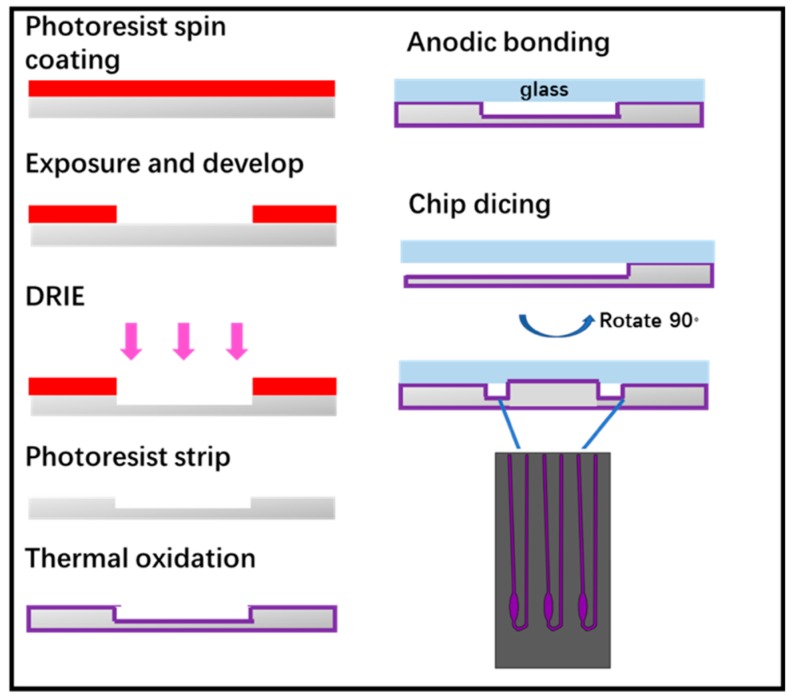
Fabrication process flow of the microchip.

The microchip was fabricated by standard lithography technology, as shown in Figure 4. Following this, 4-inch double sided polished silicon wafers were cleaned in piranha solution and hydrofluoric acid solution. The photolithographically patterned substrate first subjected to descum process in O_2_ plasma. Then, induced coupled plasma-deep reaction ion etching (ICP-DRIE) was applied to etch silicon to form wells and channels for carrying out real-time PCR (RT-PCR) reactions. After photoresist was stripped by plasma, the wafer grown thermal oxide with a thickness of 1000 Å as a passivation layer used to avoid non-specific adsorption of PCR components. The oxide wafer was bonded with a 4-inch glass wafer by anodic bonding to form a closed PCR chamber and finally the wafer was diced into individual microchips. It is worth noting that we designed the chip inlet and outlet on the side of the chip rather than its top and the inlet and outlet port could be opened just after wafer dicing. This design could avoid drilling or punching processes of the inlet and outlet port, as well as making it easier for our device to add extracted nucleic acid into the microchip. The microchip designed for this experiment has three reaction chambers each had a reaction volume of 2 μL.

### 2.5. RNA Reverse Transcription

Total RNA was reversely transcribed into cDNA with the following protocol: RNA mixture contained 2 μL of template (extracted using the automated miniaturized device as described above), 1 μL primer (Thermal Fisher Scientific, Carlsbad, CA, USA), 1 μL dNTPs (Thermal Fisher Scientific, USA) and 6 μL ddH_2_O to bring the volume up to 10 μL. The mixture was then heated to 65 °C for 5 min and the sample was quickly chilled on ice for 2 min. A reaction mixture was prepared in a new and separate tube including 4 μL 5 × buffer (Thermal Fisher Scientific, USA), 0.5 μL RT enzyme (Thermal Fisher Scientific, USA), 0.5 μL inhibitor (Thermal Fisher Scientific, USA), and 5 μL ddH_2_O to form a 10 μL solution. Following this, 10 μL reaction mixture was added to 10 μL RNA mixture then the sample was incubated at 42 °C for 60 min followed by 25 °C for 5 min. 

### 2.6. Detection of Gene Expression by Real-Time PCR

The cDNA was amplified by real-time PCR, and the amplification target genes Actin and GAPDH were verified by 2% agarose gel electrophoresis. The GAPDH primers consist of a forward primer CAT GAG AAG TAT GAC AAC GCC T and a reverse primer AGT CCT TCC ACG ATA CCA AAG T, which produce a PCR product with a size of 113 bp. The Actin primers were forward primer GAG CAC AGA GCC TCG CCT TT and reverse primer TCA TCA TCC ATG GTG AGC TGG C resulted in a PCR product with a size of 70 bp. Each RT-PCR reaction was performed in 10 μL 2 × Bio-Rad super mix, 1 μL of RT reaction as cDNA, 0.5 μL primers (10 μM) and 8.5 μL ddH_2_O. The cycling parameters involved denaturation at 95 °C for 3 min, followed by 40 cycles of 95 °C for 30 s and 60 °C for 1 min. Each target gene expression was amplified by two repeated experiments. The RT-PCR was performed using LineGene 9600 Plus (Bioer) and its cycle threshold (Ct) value was determined.

### 2.7. Materials

293T cells were cultured in DMEM (DMEM; Gibco, USA) with 10% FBS (FBS; Gibco, USA) and 1% Penicillin/Streptomycin at 37 °C in 5% CO_2_, MagMAX^TM^-96 Total RNA Isolation Kit (Thermal Fisher Scientific, Carlsbad, CA, USA), 100% isopropanol, 100% ethanol, Maxima H Minus First Strand cDNA Synthesis Kit (Thermal Fisher Scientific, USA), iQ^TM^ SYBR Green Supermix (BIO-RAD, Foster City, CA, USA), NanoDrop^TM^ One (Thermal Fisher Scientific, USA). 

## 3. Results and Discussion

**Figure 5:**
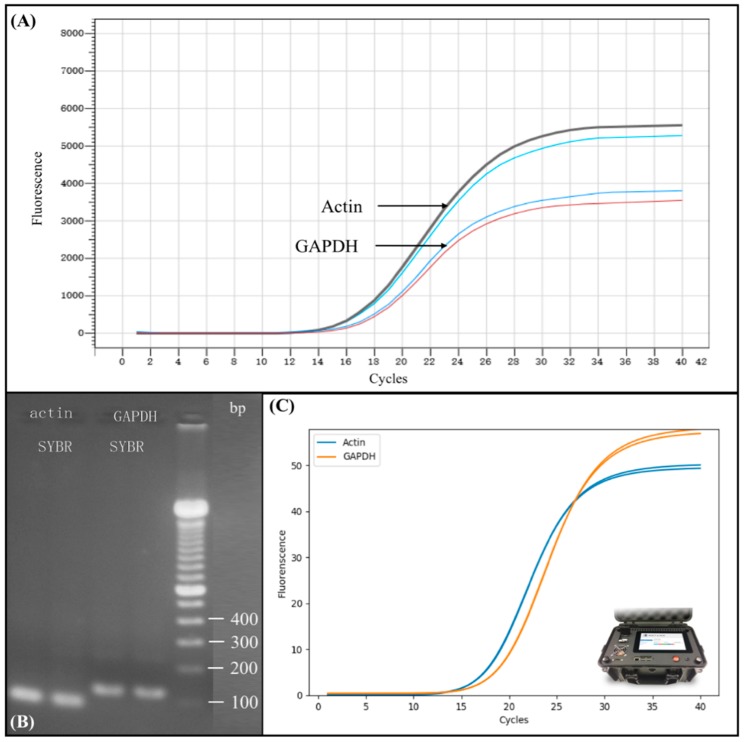
Verification of real-time polymerase chain reaction (PCR) results using total RNA extracted from 293T cells using automated miniaturized nucleic acid sample preparation device. (**A**) Real-time PCR amplification results on commercial real-time PCR instrument (BIOER) of Actin and GAPDH gene expression. (**B**) Electrophoresis gel separation and detection of Actin and GAPDH gene expression as a successful amplification result verification. (**C**) Comparable real-time PCR amplification results on portable microchip real-time PCR instrument using the same sample and protocol.

In order to test the performance of the automated miniaturized device for nucleic acid sample preparation, we conducted reverse transcript and real-time PCR using the sample obtained from the device. The results are shown in Figure 5. Prior to testing, the total RNA was extracted from 293T cells by a complete automated process using our automated miniaturized nucleic acid sample preparation device. The concentration of total RNA was quantified by NanoDrop^TM^ One and was found to be 500 ng/μL. Total RNA was then reversely transcribed into cDNA and we performed real-time PCR using the protocol as described in Materials and Methods. Since 2 μL total RNA was used in transcription, the concentration of cDNA is 50 ng/μL, thus add 1 μL cDNA into each PCR reaction is equivalent to adding 50 ng of sample into each PCR reaction. We then used Actin and GAPDH gene expression as standard in PCR amplification. Figure 5A shows the real-time PCR amplification results on commercial real-time PCR instrument (BIOER) of Actin and GAPDH gene expression. The upper black and the light blue curve represent two repeated RT-PCR amplification result of Actin, and the lower blue and red curve represent two repeated RT-PCR amplification result of Glyceraldehyde 3-phosphate dehydrogenase (GAPDH). The Ct value of Actin and GAPDH was 14 and 16, respectively, demonstrating that RNA was successfully extracted from the sample cell using completely automated process without human intervention. The Ct value also shows a rather high concentration of the extracted product. 

To verify the extracted RNA from 293T cells, we conducted a downstream analysis using conventional electrophoresis gel separation. Figure 5B shows the electrophoresis results of Actin and GAPDH gene expression as an amplification result verification. The marker we used, as shown in the far right line in Figure 5B, was Thermo Fisher Scientific 100 bp DNA Ladder. The results show that the extracted RNA via post-PCR from automated sample preparation device could be successfully separated and clearly detected using the conventional gel electrophoresis, indicating the feasibility of our device for efficient nucleic acid sample preparation.

In order to further verify the performance of our developed device, we compared the real-time PCR amplification results from large commercialized real-time PCR instruments with portable microchip real-time PCR device, as shown in Figure 5C. The amplification curve shown in Figure 5C was attained using the same total RNA extracted from our automated miniaturized device followed by reverse transcription into cDNA, as previously described. However, the DNA was amplified using another of our devices, the portable microchip real-time PCR device, as shown in Figure 5C, rather than a commercialized PCR instrument. Each PCR reaction was carried out using 2 μL PCR mixture, as previously described. The Ct value of Actin and GAPDH was 14 and 16, respectively, which was identical to the experiment results obtained from large commercial PCR instruments. These consistent results illustrate that the total RNA extracted had high purity, as even 2 μL volume of PCR mixture could demonstrate the same result, thus proving the efficiency of our automated miniaturized device. Moreover, our portable microchip real-time PCR instrument generated the same Ct value as commercialized PCR instruments.

## 4. Conclusions

In conclusion, we have developed an automated miniaturized device to achieve automation of nucleic acid purification from actual samples. This device can adapt to different extraction protocols by configuring the software in the external central controller to meet personalized needs, and the extracted nucleic acid sample could be directly introduced into the microchip for further downstream applications. This method enables an easy and time-saving nucleic acid extraction, regardless of the experience of the operator. The data indicated that the automated total RNA extraction was equivalent to the performance using a manual process, but the automated extraction is more time-saving with the whole process curtailed to 10 min. The extracted total RNA from 293T cells can be verified by either PCR with post gel electrophoresis or qPCR. The process does not require external instrument for centrifugation or precipitation ensures its portability. In the future, we can integrate the automated miniaturized device with our portable microchip real-time PCR instrument to achieve a fully automated experimental process for practical biomedical applications.
